# Right gyrus cinguli low-grade astrocytoma recurrence removed through a contralateral transfalcine approach with a 4K-3D exoscope

**DOI:** 10.1186/s41016-023-00320-9

**Published:** 2023-03-07

**Authors:** Stefano Peron, Giovanni Marco Sicuri, Andrea Cividini, Roberto Stefini

**Affiliations:** Neurosurgical Unit, Department of Neurosciences, ASST Ovest Milanese-Legnano Hospital, Via Papa Giovanni Paolo II, 20025 Legnano, Milan Italy

**Keywords:** 4K-3D images, Contralateral approach, Exoscope, Glioma

## Abstract

**Background:**

Brain tumor surgery has been using operative microscope for years. Recently, thanks to developments in surgical technology with procedures performed on head-up displays, exoscopes have been introduced as an alternative to microscopic vision.

**Case presentation:**

We present a case of a 46-year-old patient with a low-grade glioma recurrence of the right gyrus cinguli removed with a contralateral transfalcine approach using an exoscope (ORBEYE 4K-three-dimensional (3D) exoscope, Sony Olympus Medical Solutions Inc., Tokyo, Japan).

The operating room setup for this approach is illustrated.

During the procedure, the surgeon was seated with head and back in an upright position, while the camera was aligned with the surgical corridor.

The exoscope provided detailed, high-quality 4K-3D images of the anatomical structures and optimal depth perception, making surgery accurate and precise.

At the end of the resection, an intraoperative MRI scan showed complete removal of the lesion. The patient was discharged on postoperative day 4 with an excellent performance on neuropsychological examination.

**Conclusions:**

In this clinical case the contralateral approach was favorable because the glioma was located close to the midline and because it offered a straight path to the tumor, minimizing retraction on the brain. The exoscope provided the surgeon with important advantages in terms of anatomical visualization and ergonomics during the entire procedure.

**Supplementary Information:**

The online version contains supplementary material available at 10.1186/s41016-023-00320-9.

## Background

In recent years, thanks to significant developments in screen-based technology with head-up displays, exoscopes have also been introduced in surgery as a viable alternative or in addition to microscopic vision. Actually, brain tumor surgeries require a magnified view of the surgical field and anatomical details, which are traditionally provided by the microscope.

In particular, the contralateral approach is defined as one that uses a cross trajectory in a coronal plane to reach a lesion contralateral to the craniotomy. In microsurgery, the need to be minimally invasive often prevails on a shorter surgical route and a cross trajectory might sometimes be useful in order to preserve the brain during tumor removal [1].

We present a case of a 46-year-old patient with a low-grade glioma recurrence of the right gyrus cinguli removed with a contralateral transfalcine approach using an exoscope (ORBEYE 4K-three-dimensional (3D) exoscope, Sony Olympus Medical Solutions Inc., Tokyo, Japan).

The advantages of the contralateral approach, combined with the use of the exoscope, will be thoroughly illustrated and discussed.

## Case presentation

This is a 46-year-old male patient who was operated on in 2016 to remove a diffuse grade II astrocytoma of the right gyrus cinguli, through an ipsilateral interhemispheric approach with the head in neutral position. The follow-up with brain MRI showed no recurrence until April 2021, when a new MRI demonstrated a local recurrence. At that time, the patient complained only of a mild headache with no neurological deficits.

**Fig. 1:**
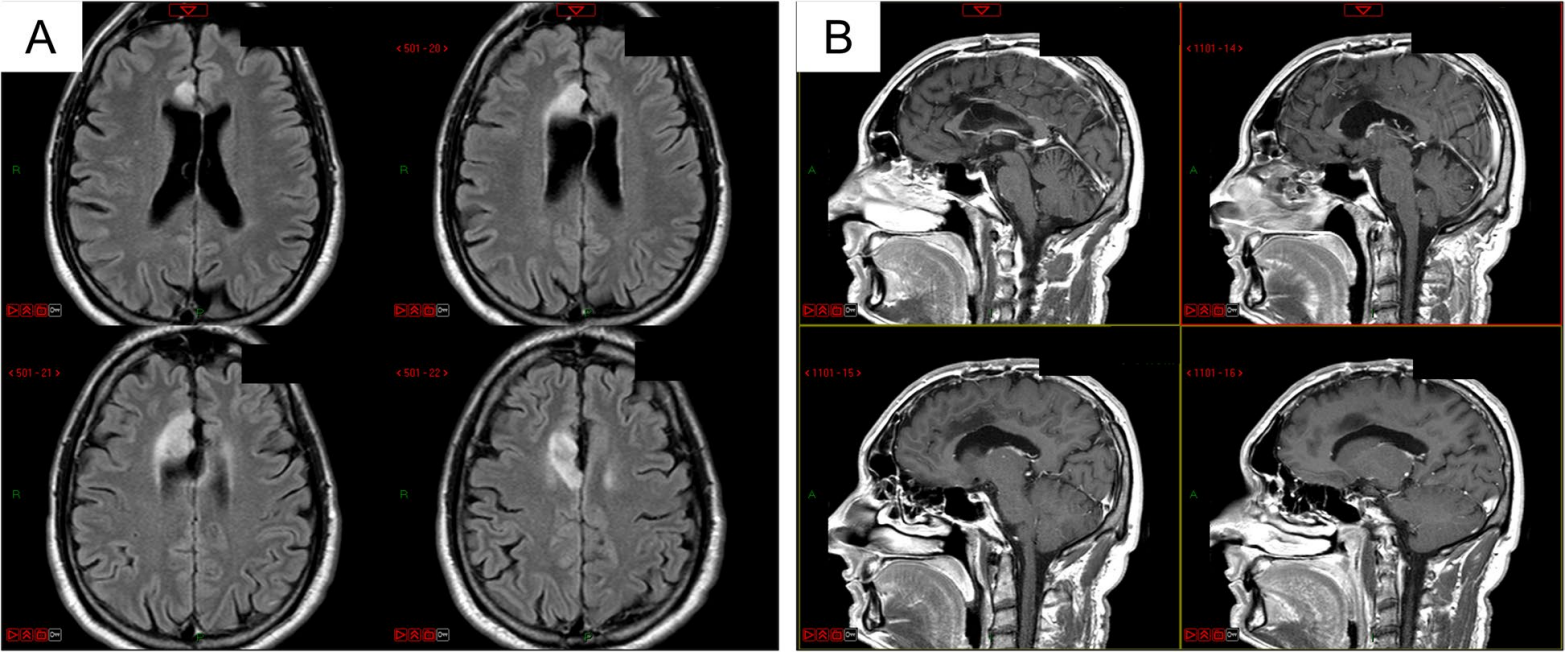
Preoperative FLAIR (**A**) and contrast enhanced T1-weighted (**B**) magnetic resonance images showing a glioma in the right cingulate gyrus

The MRI showed a FLAIR hyperintense and T1 hypointense, no-enhanced lesion at the level of the anterior third of the right gyrus cinguli, suggestive of low-grade glioma recurrence (Fig. 1).

**Fig. 2:**
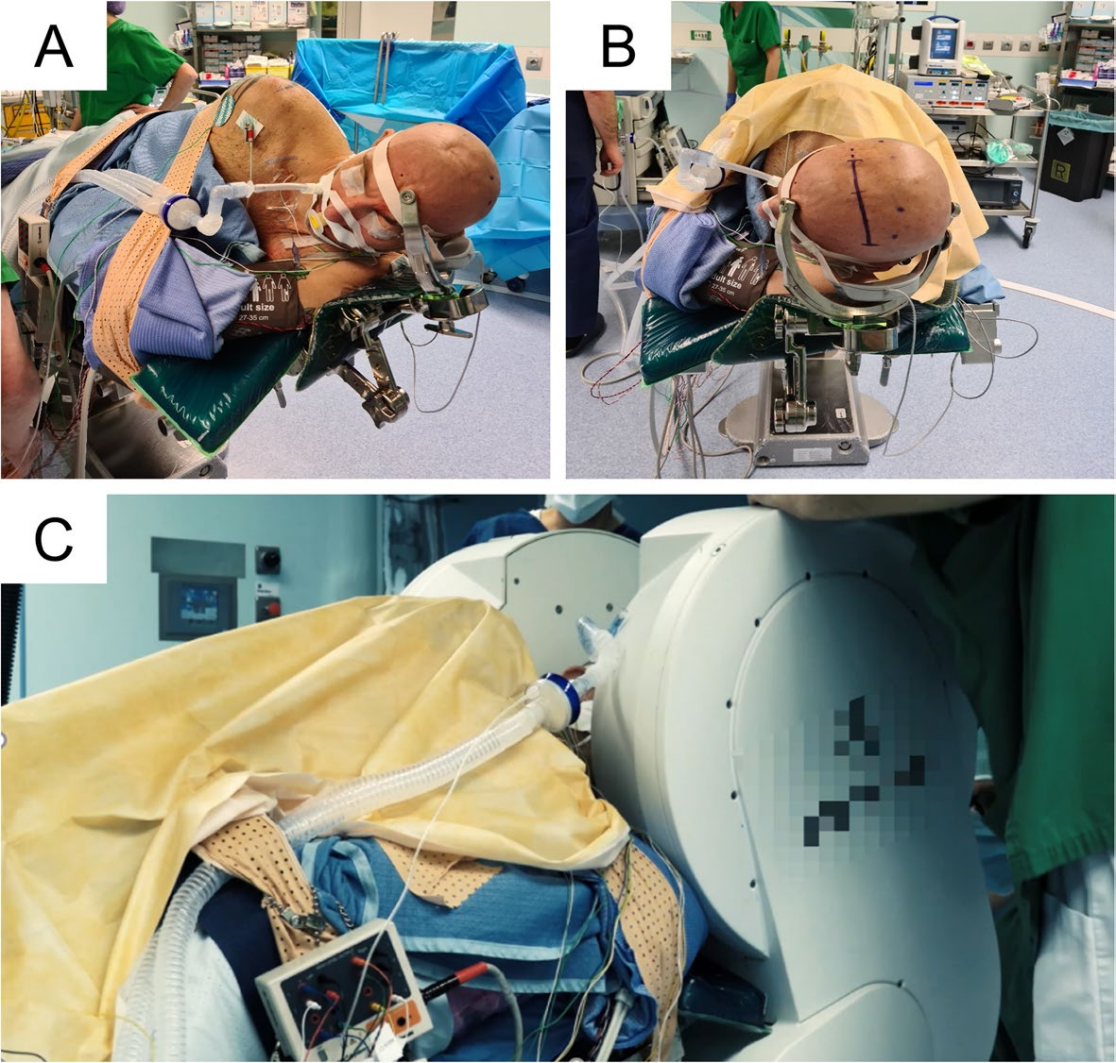
Patient positioning for contralateral transfalcine approach from left to right (**A**) and low-field MRI setup for intraoperative scanning (**B**)

Our patient was placed in a left lateral position. The head was fixed in a three-pin non-magnetic headholder with the hemisphere contralateral to the lesion below and the falx parallel to the floor. The head was angled about 45° upward to optimize the angle of view toward the right hemisphere (Fig. 2A, B).

In this surgery, we decided to use intraoperative low-field MRI for better control of the tumor resection (Fig. 2C).

**Fig. 3:**
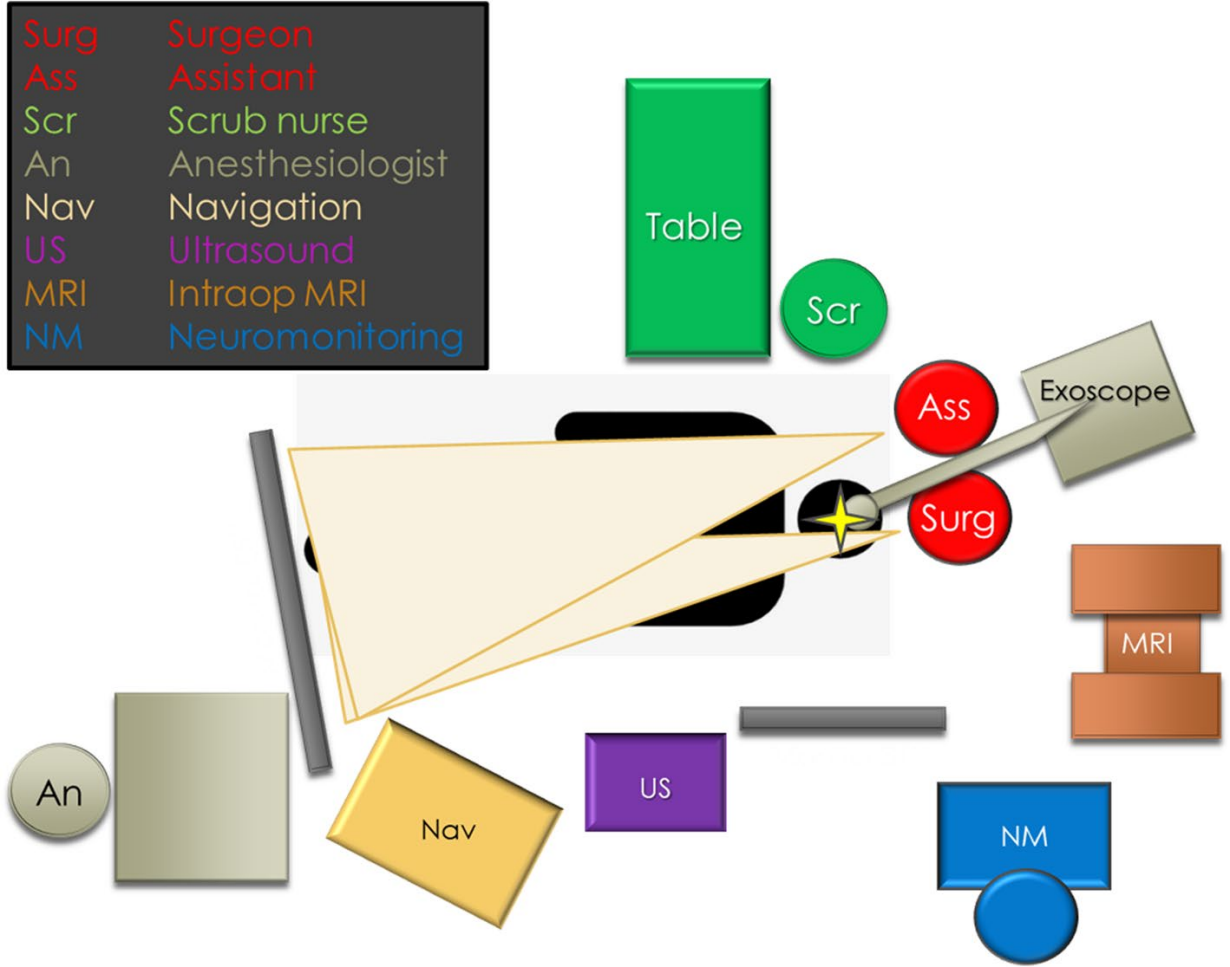
OR setup for a contralateral approach with exoscope

In addition to the 4K-3D exoscope and intraoperative MRI, a neuronavigation system, an ultrasound scanner and the neurophysiological monitoring device were set up for this procedure (Fig. 3).

**Fig. 4:**
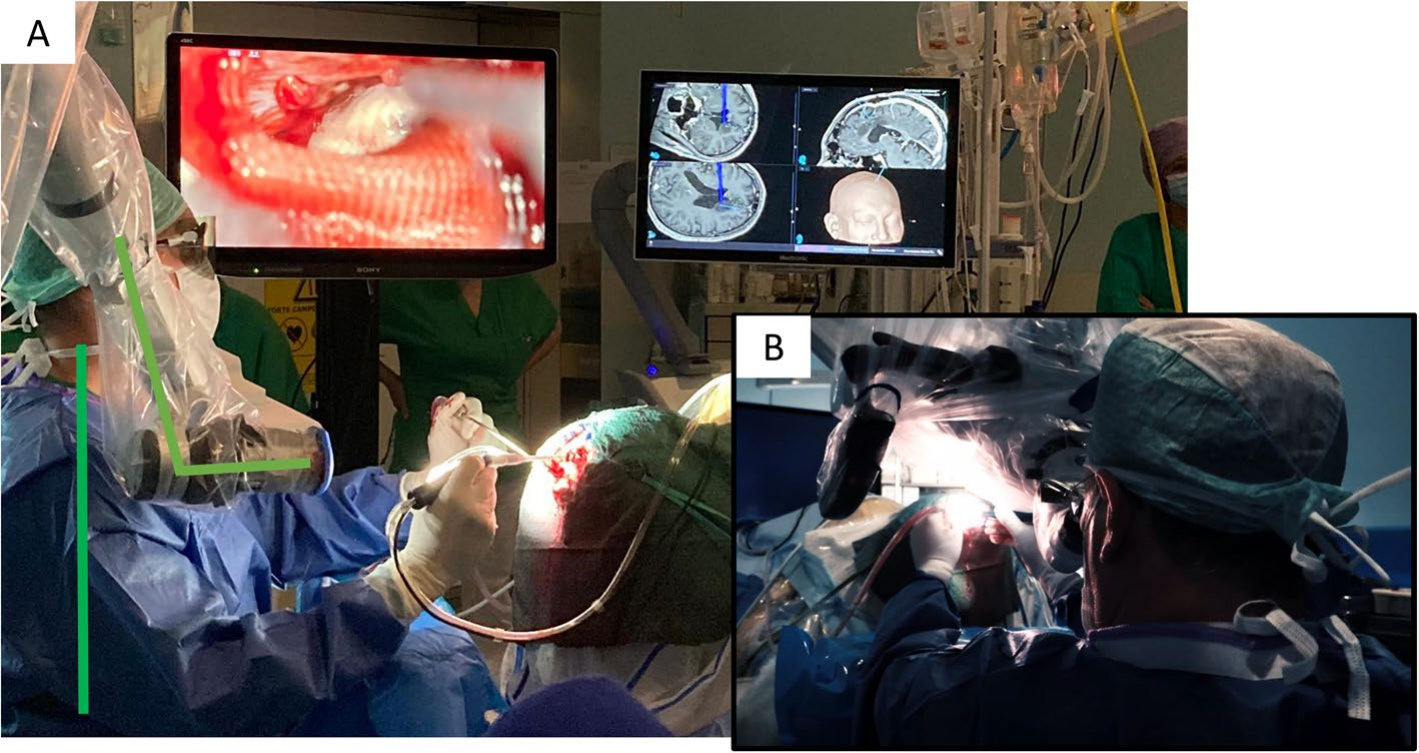
Unlike with the microscope, the surgeon can operate in a comfortable position with the exoscope aligned with the surgical corridor

During surgery, the surgeon was positioned with head, shoulders, and back in an upright position, while the orbital camera is aligned with the surgical corridor (Fig. 4).

A linear skin incision was performed in the frontal region crossing the midline followed by a craniotomy to expose the superior sagittal sinus.

**Fig. 5:**
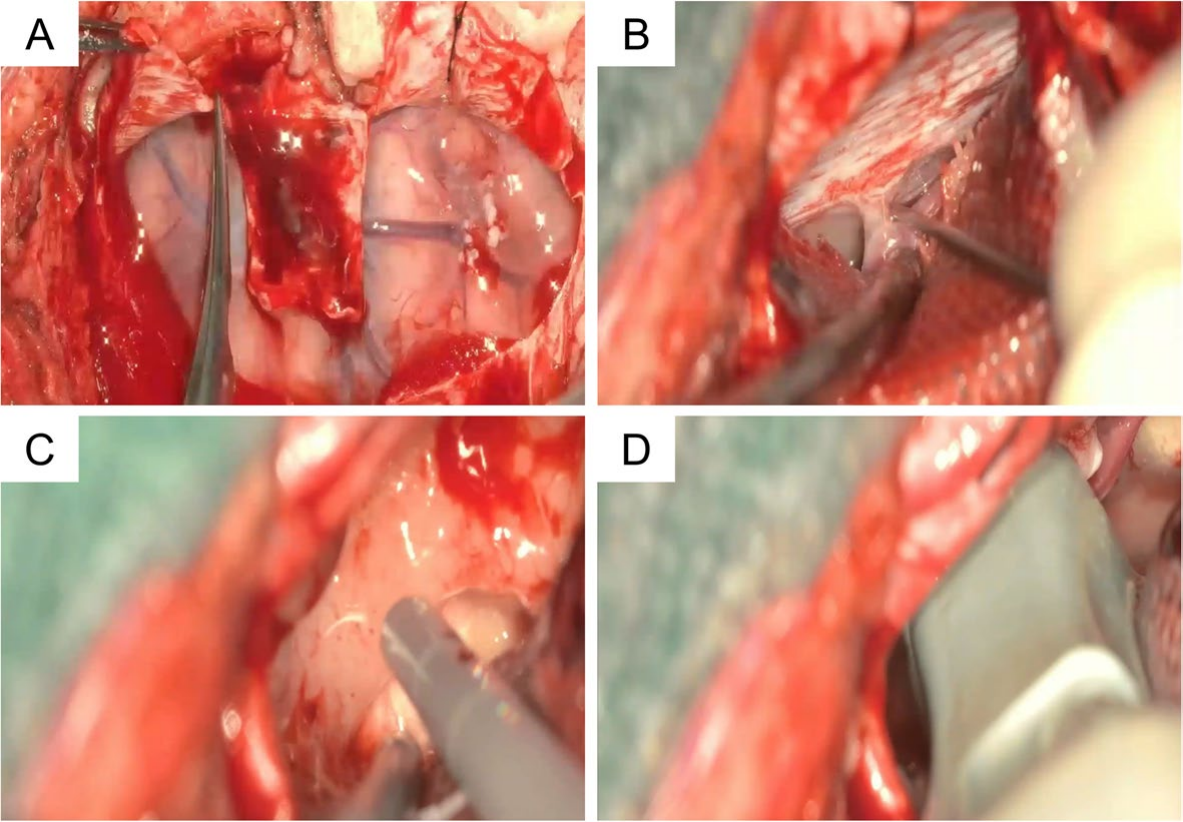
During dura opening, bridging veins must be carefully preserved (**A**); the left interhemispheric fissure is opened to reach the contralateral gyrus cinguli (**B**); the tumor is removed using an ultrasonic aspirator (**C**); before the intraoperative MRI scan at the end of removal, an ultrasound check is performed to rule out residual tumor (**D**)

From this time, the procedure continued by visualization with a 4K-3D exoscope (Fig. 5A–D). If you wear 3D polarized glasses, an additional 3D movie file shows you the OR setup and the key points of the procedure in more detail (Additional file 1).

The dura was opened with a horseshoe-shaped flap based along the sinus.

Tack-up sutures were performed for greater rotation of the sagittal sinus and better exposure of the interhemispheric fissure, the falx, and contralateral gyrus cinguli.

The falx was then cut for wider exposure of the tumor.

An intraoperative ultrasound check with a high-frequency straight probe was performed to assess the limits of the tumor.

In addition, a blue light filter was used to check the 5-ALA tumor metabolism, with no fluorescence to further confirm the low malignancy of this glioma. The corpus callosum were clearly visible inferior to the tumor.

Finally, the tumor could be easily removed using an ultrasonic aspirator. During removal, ultrasound was frequently used to rule out any residual tumor. At the end of the procedure, no more tumor was visible.

**Fig. 6:**
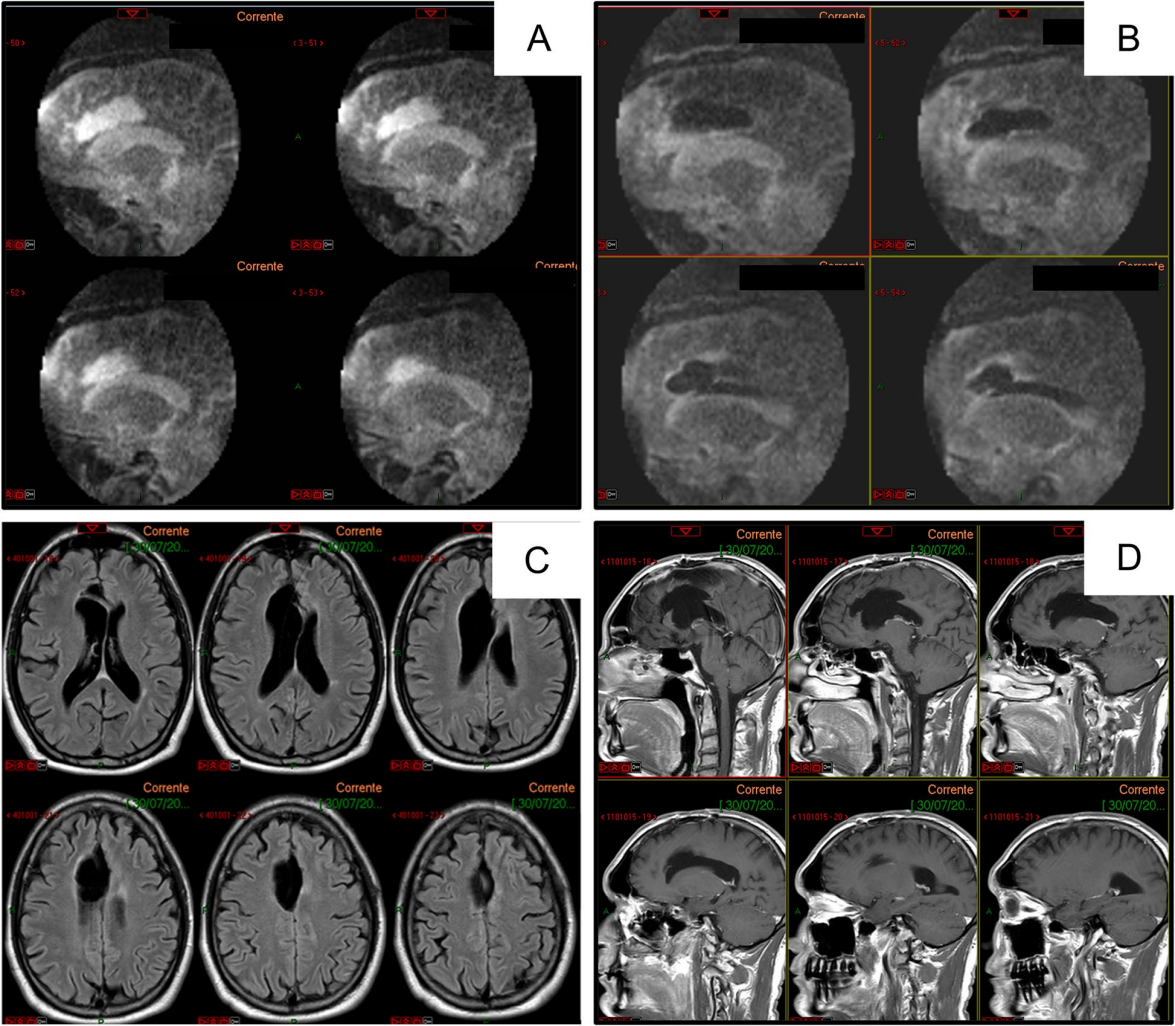
Intraoperative MRI scan showing complete removal of the lesion, as demonstrated comparing the T2-weighted sequences before (**A**) and after (**B**) surgery; three months later FLAIR (**C**) and contrast enhanced T1-weighted (**D**) magnetic resonance images showing no recurrence

At the end of the resection, the iMRI scan showed complete removal of the lesion (Fig. 6A, B).

Histological and molecular examinations concluded IDH mutant 1p/19q non-codeleted diffuse grade II astrocytoma. Adjuvant therapy was not deemed necessary based on the histology and extent of removal.

The patient’s clinical outcome was optimal, with no postoperative deficits and excellent surgical wound healing.

The patient was discharged on postoperative day 4 with an excellent performance on neuropsychological examination, which was then repeated three months later with the same results.

Three months later, a new MRI found no residual or recurrent tumor (Fig. 6C, D).

## Discussion and conclusions

As a rule of thumb, the ideal approach to remove a brain tumor should provide a straight path to the lesion with the shortest working distance and the largest working angle, without jeopardizing the functional cortex or white matter tracts.

In this case, we decided to remove the glioma of the right cingulate gyrus using a contralateral transfalcine approach, exactly from left to right. Therefore, the new surgical corridor was not affected by the ipsilateral interhemispheric approach used in the first surgery.

This approach is favorable because the glioma is located close to the midline and because it offers a direct corridor to the tumor, taking advantage of gravity and minimizing retraction on the brain ipsilateral to the lesion [1].

A contralateral vein damage with ipsilateral neurological deficits, with respect to the lesion, is one of the main risks of such a surgical approach. When the dura opening is performed, all efforts should be made to preserve bridging veins in order to avoid venous ischemic complications. In fact, the number of veins draining into the superior sagittal sinus between the coronal and the lambdoid suture is considerably greater than that before the coronal or after the lambdoid suture.

In addition, some difficulties in reaching and managing the lateral aspect of the tumor might be encountered and a longer trajectory, compared with the ipsilateral approach, may require longer surgical instruments.

On the other hand, the contralateral approach offers unquestionable advantages, including no or minimal traction on the ipsilateral brain without fixed retractors along with direct and wide exposure of the tumor.

A viable alternative to this approach is the ipsilateral interhemispheric approach, with the patient in a supine or lateral position. However, this approach requires more traction on the ipsilateral brain, which could be swollen and fragile, as in the case of malignant tumors. In addition, with the head in a neutral position, the ipsilateral approach loses the advantages of gravity. For information, in the case of vertical orientation of the exoscope, the camera can be kept away from the operating field, taking advantage of the maximum focal distance (i.e., 550 mm) and maximum magnification power provided by the equipment. In this way, the camera does not interfere with the surgeon’s line of sight.

In general, lesions close to the midline typically require less retraction in order to control the lateral aspect of the surgical cavity, and they could also be accessed using an ipsilateral interhemispheric approach. Conversely, eccentric lesions require more retraction, with an increased risk of retraction-related injuries and residual disease.

**Fig. 7:**
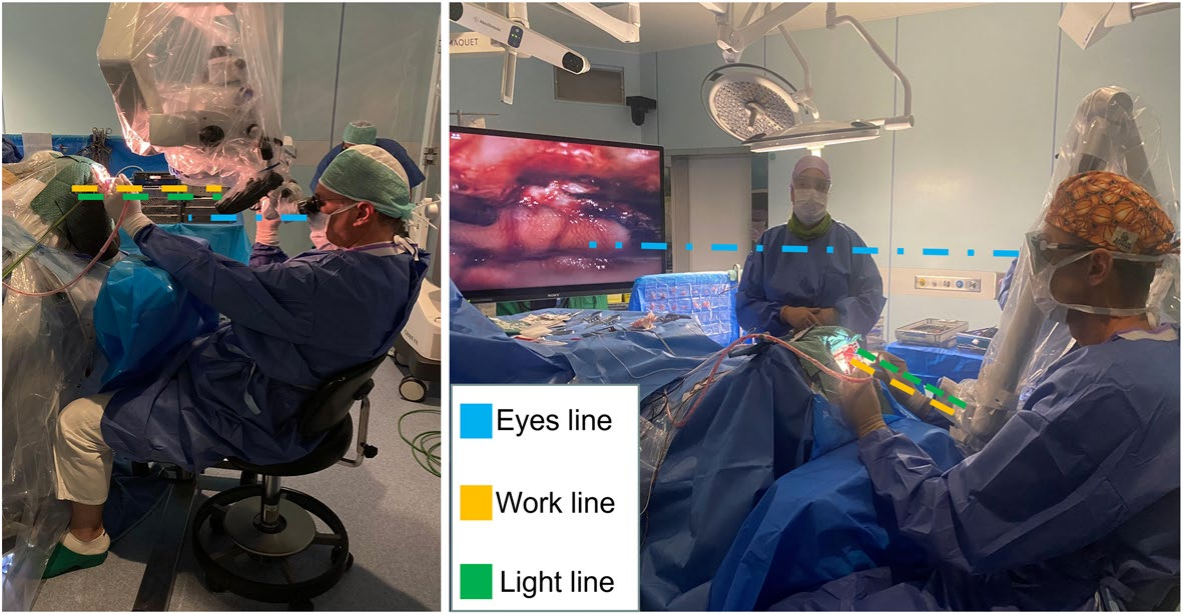
Unlike the microscope, in contralateral approaches the exoscope allows the surgeon to operate while keeping a line of sight other than the line of light/work

Until now, we were always used to thinking that the position of the head was critical to ensure good exposure of the lesion, avoiding brain retraction or manipulation. The exoscope attempts to twist this well-established concept. In fact, with the microscope the line of work/light should be the same as the line of sight, unless you want to hold an uncomfortable working position with your neck bent over the lenses. With the exoscope, the line of sight can be fully different from the line of work/light and the surgeon can operate in an ergonomic position with the head upright and good alignment of the shoulders and back [2, 3] (Fig. 7). Conversely, in a contralateral approach, the surgeon is forced to maintain an unfavorable position when using the microscope, leading to arm and shoulder fatigue (Fig. 4).

Moreover, the high-resolution 4K-3D images provided by the exoscope make surgery more accurate, with optimal perception of depth, colors, and magnification [4–6].

Furthermore, the exoscope allows more space around the operating table and patient. This is especially useful in procedures where multiple equipment is required, for example navigation devices or ultrasound, as in our surgical case.

It should not be forgotten that the exoscope offers high quality, 4K-3D images not only for surgeons, but for everyone who watches the 55-inch or 31-inch monitor using polarized glasses. This advantage can also be used for educational purposes for students and residents, because the use of external monitors and glasses gives them the same high-resolution view as the surgeon.

To conclude, in case of glioma located close to the midline, the contralateral approach is favorable because it offers a straight path to the tumor, minimizing retraction on the brain. Moreover, using an exoscope, the surgeon is seated with head and back in an upright position, while the camera is aligned with the surgical corridor. This offers unquestionable advantages in terms of comfort for the surgeon and safety for the patient.

## Supplementary Information

**Additional file 1.** The advantages of a contralateral approach, combined with the use of the exoscope, in a patient with a low-grade glioma recurrence of the right gyrus cinguli can be appreciated in this 3D video.

## Data Availability

Not applicable.

## Abbreviations

4k-3D: 4k-three dimensional
OR: Operating room
MRI: Magnetic resonance imaging
